# MELD Scores and Child–Pugh Classifications Predict the Outcomes of ERCP in Cirrhotic Patients With Choledocholithiasis: A Retrospective Cohort Study

**DOI:** 10.1097/MD.0000000000000433

**Published:** 2015-01-26

**Authors:** Jinshun Zhang, Liping Ye, Jinlan Zhang, Minhua Lin, Saiqin He, Xinlin Mao, Xianbin Zhou, Fachao Zhi

**Affiliations:** From the Guangdong Provincial Key Laboratory of Gastroenterology (Jinshun Z, FZ), Department of Gastroenterology, Nanfang Hospital, Southern Medical University, Guangzhou; and Department of Gastroenterology (Jinshun Z, LY, Jinlan Z, ML, SH, XM, XZ), Taizhou Hospital, Linhai, Zhejiang, China.

## Abstract

Endoscopic retrograde cholangiopancreatography (ERCP) is challenging in cirrhotic patients with choledocholithiasis. We evaluated the safety and efficacy of ERCP in cirrhotic patients with choledocholithiasis and accessed the model for end-stage liver disease (MELD) scores and Child–Pugh classifications for prediction of morbidity and mortality.

From January 2000 to June 2014, 77 ERCP operations were performed in cirrhotic patients with choledocholithiasis. The data on operative complications were analyzed. MELD scores and Child–Pugh classifications were calculated and associated with operative outcomes and survival. Telephone follow-up was performed to determine survival situations.

No death, perforation, or hemorrhage caused by gastroesophageal varices occurred as a result of the procedure. The rate of intraoperative hemorrhage was 13.0%, and the rate of postoperative morbidity was 27.3% including hemorrhage (18.2%), post-ERCP pancreatitis (6.1%), aggravated infection of the biliary tract (1.3%), hepatic encephalopathy (1.3%), and respiratory failure (1.3%). Four (5.2%) patients had both intraoperative and postoperative hemorrhage. Receiver operating characteristic analysis identified MELD scores higher than 11.5 as the best cutoff value for predicting complication incidence (95% confidence interval = 0.63–0.87). Twenty-one (44.7%) patients with a MELD score above 11.5 developed a complication, and 3 (10%) patients who had a lower MELD score developed a complication (*P* = 0.001). Both MELD score and Child–Pugh classification had prognostic value in patients without jaundice, although sex may result in different prognostic values based on the 2 scores. The rate of complications was not significantly different among patients with different Child–Pugh classifications. No significant difference was observed in patients with different MELD scores or Child–Pugh classifications in terms of median survival times.

ERCP is an effective and safe procedure in cirrhotic patients with choledocholithiasis. MELD scores can predict the risk of operative complications, but Child–Pugh classification system scores do not predict the risk of complications.

## INTRODUCTION

The incidence of gallstones has been found to be 29.4% in cirrhotic patients and 12.8% in the noncirrhotic population.^1^ The frequency of choledocholithiasis in cirrhotic patients is 3 times higher than that in noncirrhotic patients.^2,3^ Acute cholangitis and acute obstructive suppurative cholangitis (AOSC) have been reported in 44% and 19% of cirrhotic patients with choledocholithiasis, respectively.^4^ Endoscopic retrograde cholangiopancreatography (ERCP) has become the first choice for treatment of choledocholithiasis. The main complications, such as hemorrhage, associated with this procedure are unavoidable because they are caused by endoscopic sphincterotomy (EST) and/or endoscopic papillary balloon dilatation (EPBD). Coagulation dysfunction, liver function disorder, gastroesophageal varices, and ascites may increase the risk of complications in cirrhotic patients. Mortality rates have been shown to be significantly higher in the cirrhotic population than in control because of hemorrhage.^5^

ERCP has been recommended as the initial choice of management for choledocholithiasis in cirrhotic patients despite the higher complications and mortality rates when compared with the general population.^5–9^ However, these reports included only a small number of cases. Therefore, larger sample sizes and more studies are necessary to determine a solid conclusion. Furthermore, no indicator has been investigated to predict morbidity related to this procedure.

Model for end-stage liver disease (MELD) scores have been recently used to predict postoperative outcomes for cirrhotic patients undergoing surgical or laparoscopic procedures^10–12^; however, no studies have evaluated ERCP. The Child–Pugh classification system, first used to evaluate liver function in cirrhotic patients, has been used to predict the risk of death and complications in these patients.

We retrospectively analyzed the data of 77 consecutive cirrhotic patients with choledocholithiasis who underwent ERCP and evaluated the ability of MELD scores and Child–Pugh classification scores to predict the outcomes. To our knowledge, this study included the largest sample size to date and predicted the complication outcomes for ERCP in cirrhotic patients with choledocholithiasis with MELD scores for the first time.

## METHODS

**Figure 1:**
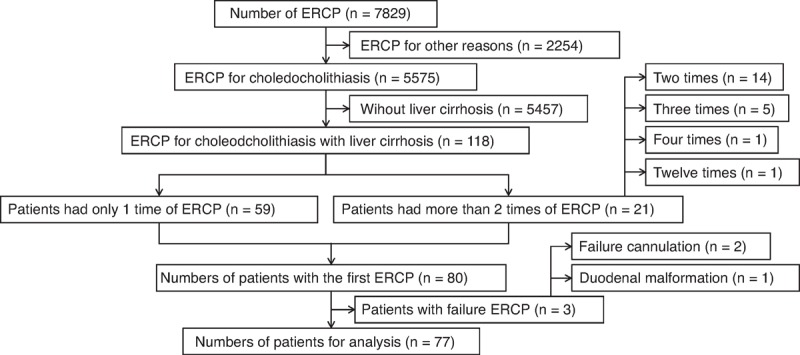
Flow diagram of study selection for cirrhotic patients with choledocholithiasis who underwent endoscopic retrograde cholangiopancreatography (ERCP). ERCP = endoscopic retrograde cholangiopancreatography.

Between January 2000 and June 2014, 7829 ERCP procedures were performed in Taizhou Hospital of Zhejiang Province, China. In total, 77 consecutive cirrhotic patients with choledocholithiasis who underwent ERCP for the first time were enrolled in this study (Figure 1). The diagnosis was based on the clinical history, physical examination, outcomes of abdominal ultrasonography and/or CT scan, and endoscopic findings. It was a retrospective study. We only collected the data of the patients, so the ethical approval was not necessary.

MELD scores were calculated using preoperative values of 3 laboratory tests: international normalized ratio (INR), serum total bilirubin (TBil), and serum creatinine (Cr). The following formula was used for calculating the MELD score: MELD = 9.57 × log_e_(Cr [mg/dL]) + 3.78 × log_e_(TBil [mg/dL]) + 11.2 × log_e_(INR) + 6.43.^13^ Child–Pugh scores were calculated using prothrombin time (PT), albumin, bilirubin, clinical findings of encephalopathy, and ascites. Child–Pugh classifications were defined as class A (5–6), class B (7–9), and class C (10–15).^14,15^

### Preoperative Preparations

Patients with coagulopathy (INR > 1.8) received fresh-frozen plasma and platelet transfusions were performed for the patients with platelet counts less than 30,000/mm^3^. Vitamin K_1_ (30 mg) was routinely administered by intramuscular injection in cases with INR > 1.8 or PT > 18. The patient or the patient's family signed an agreement after being informed of the detailed risk and preventive measures.

### Intraoperative Management

Diazepam (5 mg), pethidine (50 mg), and scopolamine (40 mg) were routinely administered by intramuscular injection in patients with Child–Pugh classification A or B, but only pethidine and scopolamine at the same dose were administered for patients with classification C to prevent hepatic encephalopathy.

The diameter of the common bile duct, the size and quantity of the stones, the patient's condition, and the Child–Pugh classification were the decisive factors for ERCP. EPBD was performed if the size of the stone was ≤1 cm, and EST if the size was >1 and <2 cm. A plastic biliary stent or endoscopic nasobiliary drainage (ENBD) was performed for stones that were ≥2 cm in size, presence of multiple stones, or Child–Pugh classification C. ENBD was performed for patients with AOSC.

Local spraying of norepinephrine or thrombin powder was used for visible hemorrhaging, which was defined as an intraoperative hemorrhage.

### Postoperative Management

Somatostatin was administered in patients with gastric and/or esophageal varices, and all patients fasted for at least 24 hours. Postoperative hemorrhage criteria were the following: observation of hematemesis and/or melena, level of postoperative hemoglobin decreased by over 2 g/dL compared with the preoperative level, or requirement of transfusion therapy. Post-ERCP pancreatitis (PEP) criteria were the following: typical pancreatic pain without perforation and the level of amylase increased to at least 3-fold over the normal level after the procedure.^16^

One author performed telephone follow-up interviews to determine the survival situation on July 11, 2014 and July 15, 2014.

### Statistical Methods

Statistical analysis was performed using IBM SPSS software, version 19.0 (IBM, Armonk, NY). Associations involving parametric data were assessed using Student *t* test. Dichotomous nonparametric data were assessed using the χ^2^ test or Fisher exact test. Coordinate points of a receiver operating characteristic (ROC) curve were used to identify significant MELD score points. The Kaplan–Meier method with a log-rank test comparison was used for survival analysis. *P* < 0.05 was considered statistically significant.

## RESULTS

In total, 77 cirrhotic patients with choledocholithiasis were enrolled in the study, and the population and clinical characteristics are shown in Table 1. Thirteen patients had a preoperative cholecystectomy because of gallbladder stones, and the rest (1/14) had a procedure for gallbladder polyps. The total incidence rate of gallbladder stones was 54.5% (42/77) in cirrhotic patients with choledocholithiasis.

**Table 1:**
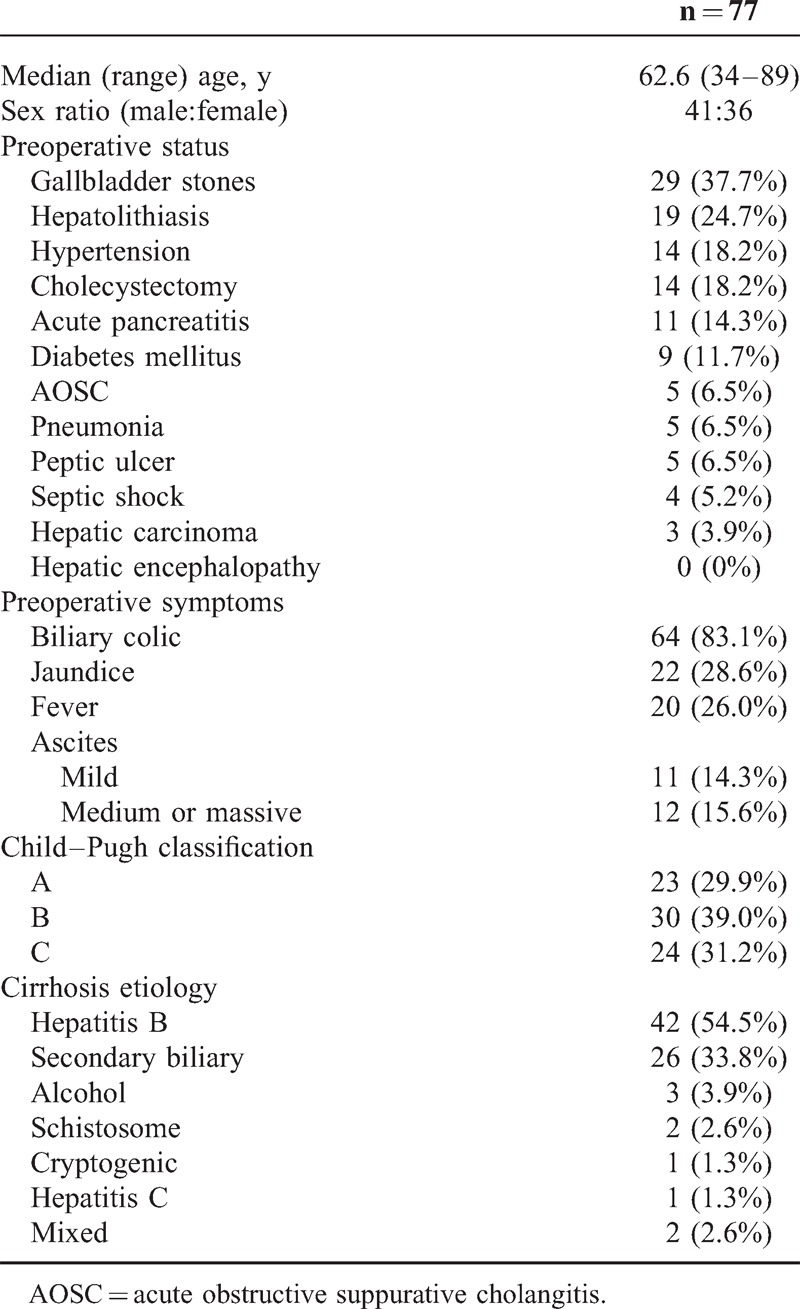
Clinical Characteristics of the 77 Cirrhotic Patients With Choledocholithiasis

The type of ERCP operation, intraoperative and postoperative complications, and blood transfusion rates are shown in Table 2. Four (5.2%) patients had both intraoperative and postoperative hemorrhage, and 75% (3/4) underwent an EST operation. The rate of intraoperative hemorrhage was not significantly different between patients receiving EST and those not receiving EST (12.5% vs 13.5%, *P* > 0.05), and the outcomes were similar for postoperative hemorrhage (22.5% vs 13.5%, *P* > 0.05). Excluding patients with preoperative acute pancreatitis, the rate of PEP was 16% (4/25) in patients with EPBD and 0% (0/31) in patients with EST (*P* = 0.034). Only 9.1% (2/22) of patients without jaundice had operative complications, whereas 40.0% (22/55) of patients with jaundice had operative complications (*P* = 0.008). No perforation or hemorrhage caused by gastroesophageal varices was observed in any case.

**Table 2:**
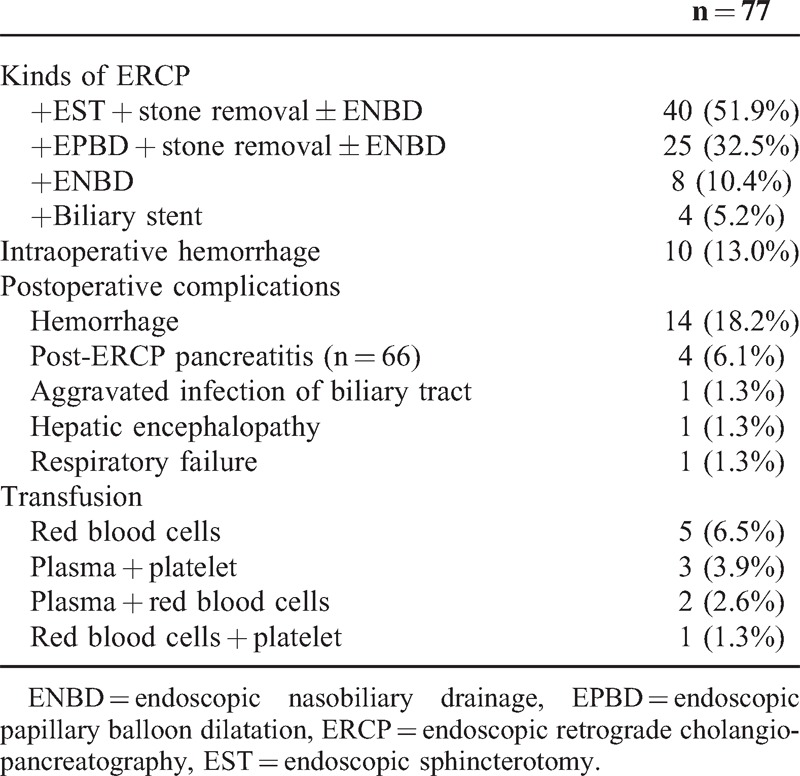
Operative Characteristics and Transfusion of the 77 Cirrhotic Patients With Choledocholithiasis

**Figure 2:**
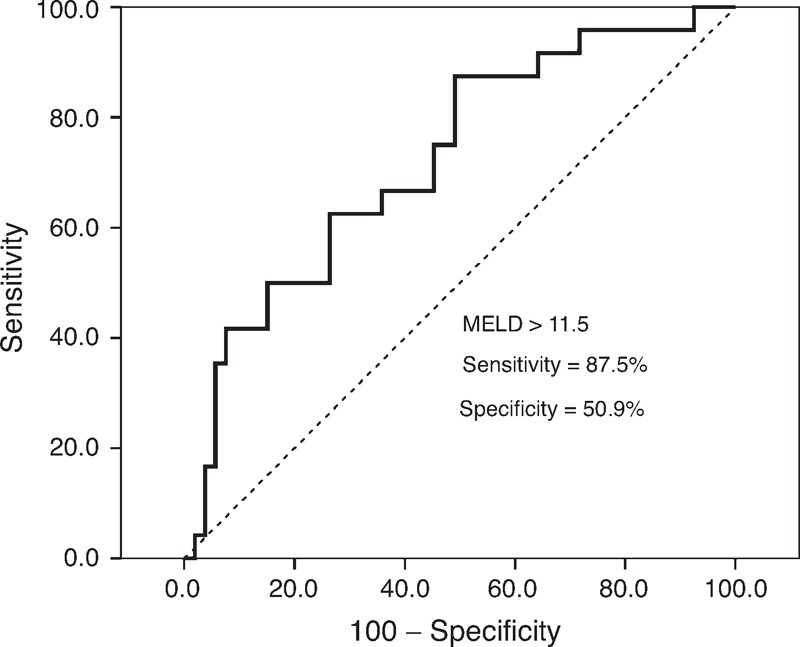
The ROC curve of MELD scores for predicting the incidence of intraoperative and postoperative complications after ERCP in cirrhotic patients with choledocholithiasis (AUC = 0.75, 95% CI = 0.63–0.87). AUC = area under the curve, CI = confidence interval, ERCP = endoscopic retrograde cholangiopancreatography, MELD = model for end-stage liver disease, ROC = receiver operating characteristic.

ROC analysis identified MELD scores higher than 11.5 as the best cutoff value for predicting the incidence of complications (area under the curve [AUC] 0.75, 95% confidence interval [CI] = 0.63–0.87; sensitivity = 87.5%; specificity = 50.9%) (Figure 2). In total, 21 (44.7%) cases developed a complication among patients with MELD scores higher than 11.5, and 3 (10%) cases with a lower MELD score developed a complication (*P* = 0.001). PEP, intraoperative hemorrhage, and postoperative hemorrhaging occurred in 2 patients with MELD scores of 23 and 21, respectively. PEP and postoperative hemorrhaging occurred in 2 patients with MELD scores of 24 and 14, respectively. Intraoperative and postoperative hemorrhaging occurred in 1 patient with a MELD score of 12. Therefore, the rate of multiple complications was higher in patients with a MELD score higher than 11.5 than in those with a score lower than 11.5 (*P* = 0.007). The rate of intraoperative hemorrhage was higher in patients with a MELD score higher than 11.5 (*P* = 0.005). Additionally, the average length of postoperative stay was significantly longer in patients with a higher MELD score (*P* = 0.035). However, no significant difference in the rate of complications was observed among patients with different Child–Pugh classifications (Table 3).

**Table 3:**
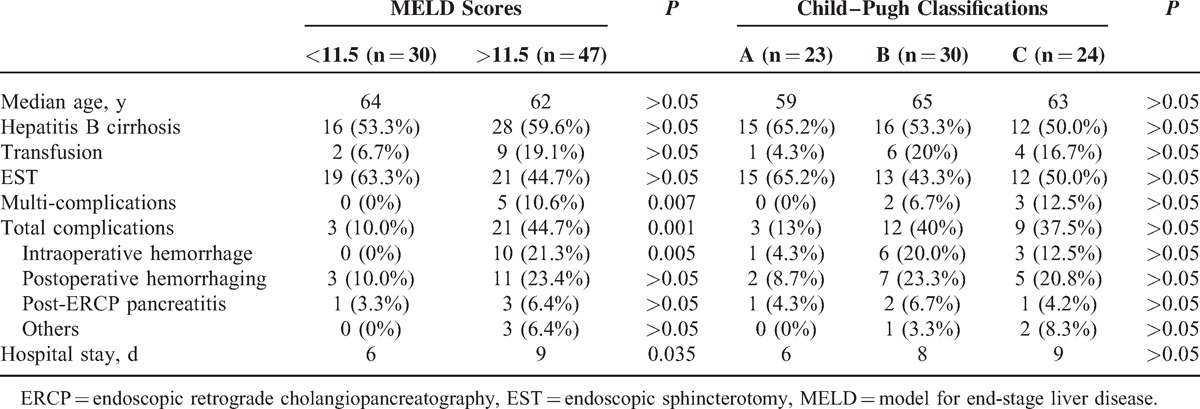
Perioperative Variables and Their Association With MELD Scores and Child–Pugh Classification System

The prognostic value of MELD scores and Child–Pugh classification scores according to sex and presence of jaundice were also assessed (Table 4).

**Table 4:**
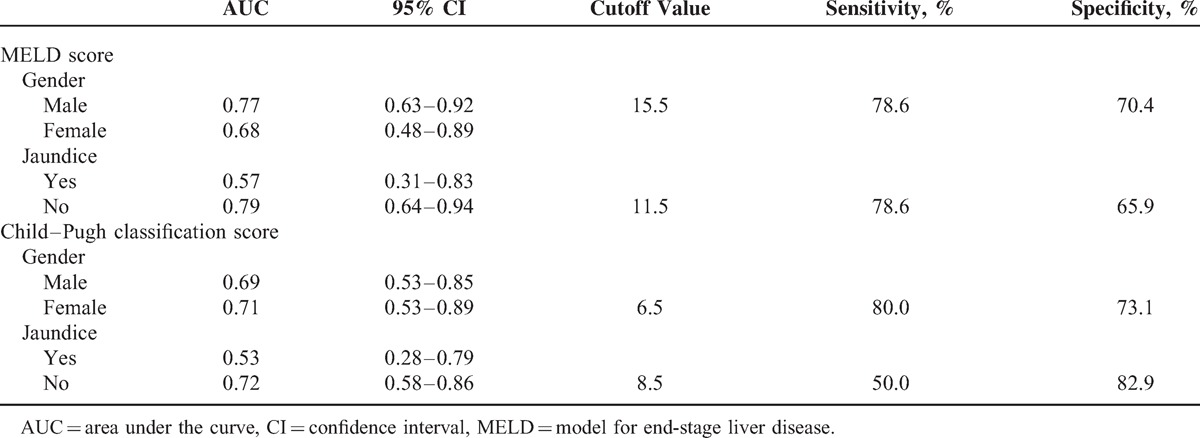
The Prognostic Value of MELD Scores and Child–Pugh Classification Scores According to Sex and Presence of Jaundice Were Also Assessed

**Figure 3:**
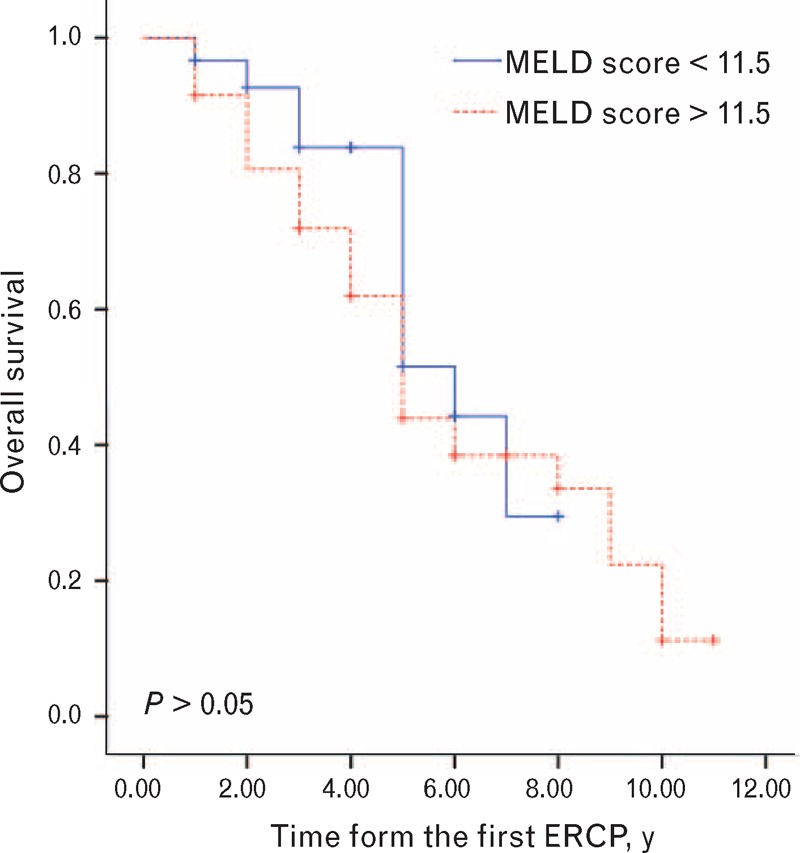
Kaplan–Meier survival curves in cirrhotic patients with choledocholithiasis according to MELD scores. MELD = model for end-stage liver disease.

**Figure 4:**
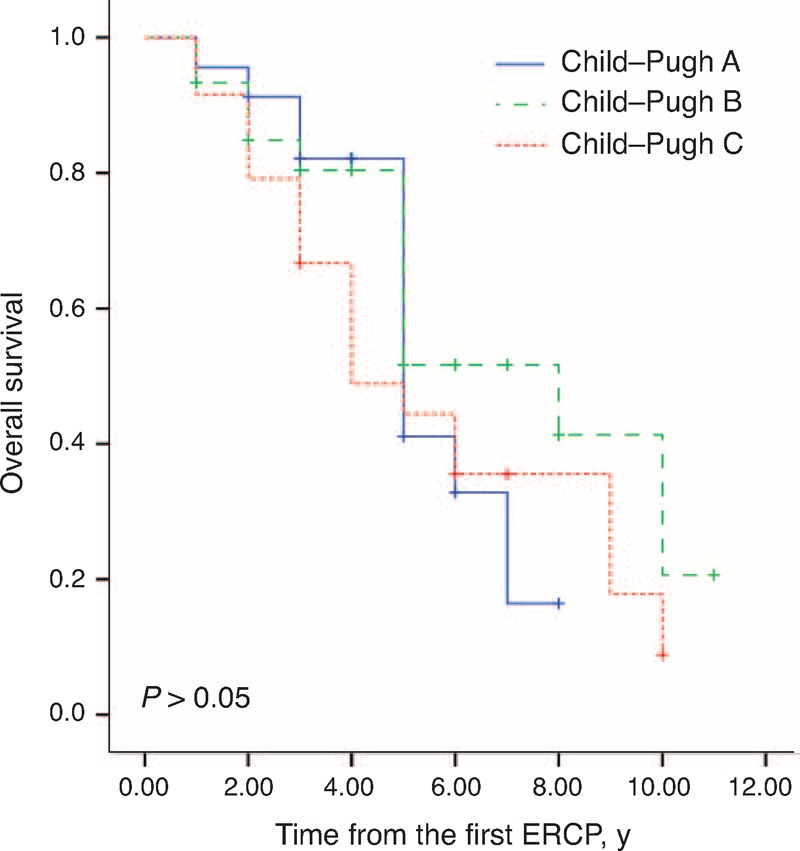
Kaplan–Meier survival curves in cirrhotic patients with choledocholithiasis according to the Child–Pugh classification system.

Twelve (15.6%) patients were lost to follow-up, and the 5-year survival rate was 47% for all patients. The Kaplan–Meier curves between the higher MELD scores (>11.5) and lower scores (<11.5) are shown in Figure 3. The Kaplan–Meier estimates of the 5-year overall survival rate in patients with higher scores and lower scores were 43.9% and 51.6%, respectively. The median survival time was 6 years (95% CI, 4.1–7.9) in patients with lower scores and 5 years (95% CI, 4.2–5.8) in patients with higher scores (*P* = 0.391). Figure 4 shows that the estimated 5-year overall survival in patients with Child–Pugh classifications A, B, and C was 41.1%, 51.7%, and 44.4%, respectively. The median survival time for patients with Child–Pugh classifications A, B, and C was 5 years (95% CI, 3.8–6.2), 8 years (95% CI, 4.7–11.2), and 4 years (95% CI, 2.1–5.8), respectively (*P* = 0.427).

## DISCUSSION

Because of their poor hepatic reserve, surgical treatment of choledocholithiasis in cirrhotic patients has high morbidity (66.7%) and mortality rates (44.4%).^4^ Laparoscopic common bile duct exploration may be effective in these patients with a Child–Pugh classification of A or B, but the conversion from laparoscopic to open surgery is unavoidable with hemorrhaging and severe adhesions.^17^ As a minimally invasive technique, ERCP has become the first choice for choledocholithiasis in the general population, and has also been reported to be useful in a few cases of cirrhosis.^5,6^ Endoscopic treatment has some advantages because several types of endoscopic technologies can be alternatives during an operation, recovery is rapid because the incision is small or absent, there is little restriction from ascites, and there is no risk associated with general anesthesia because only a sedative is used. Cirrhotic and noncirrhotic patients treated for choledocholithiasis showed similar results for stone clearance, morbidity, and mortality.^9^

However, studies have shown that the mortality and morbidity rates after EST are high,^6^ and long-term follow-up studies are needed to confirm these results.^18^ Preoperative decision making would be facilitated if the expected intraoperative and postoperative morbidity could be preoperatively predicted. Studies have shown that MELD scores are predictors for mortality after major surgery in cirrhotic patients^19^ and appear to predict morbidity more accurately than the Child–Pugh classification system in cirrhotic patients who undergo laparoscopic cholecystectomy.^12^

In this study, the rates of multiple complications, total complications, and intraoperative hemorrhage were significantly increased in patients with MELD scores higher than 11.5 (*P* = 0.007, 0.001, and 0.005, respectively). Patients with MELD scores lower than 11.5 required shorter hospital stay days after the procedure (*P* = 0.035). All other complications with a high death risk included aggravated infection of the biliary tract, hepatic encephalopathy, and respiratory problems in patients with higher MELD scores, although no significant differences were observed (*P* > 0.05). The Child–Pugh classification system could not predict a significant difference in complications or duration of hospital stay. Therefore, patients with MELD scores higher than 11.5 should be treated carefully. We suggest the following: sufficient preoperative discussion of a detailed plan for each patient, avoiding emergent operations except for significant indications, performing ENBD for life-threatening situations and waiting for the second operation, effective measures for coagulopathy, and operation by experienced endoscopists.

Both the MELD score and Child–Pugh classification had a prognostic value in patients without jaundice but not in those with jaundice. A high level of TBil represents an acute severe situation or coagulation disorder in cirrhotic patients with choledocholithiasis. The high rate of complications (40.0%) may influence the prognostic value for both scores. Additionally, MELD scores had prognostic value only in male patients, whereas Child–Pugh classification had prognostic value only in female patients (Table 4). Mindikoglu et al considered that lower liver transplantation rates among women on a waiting list could be explained in part by lower MELD scores in comparison with men as a result of lower levels of Cr in women.^20^ However, the AUC values were very close to 0.70 for MELD scores in female patients and Child–Pugh classifications in male patients (0.68 and 0.69, respectively) in our study. We therefore consider that sex may impact the prognostic value of both scores, but more studies are needed.

MELD scores were a reliable measure of mortality risk within 3 months in patients with end-stage liver disease.^21^ Our study shows a lack of significant difference in patients with different MELD scores or Child–Pugh classifications in terms of median survival time (*P* = 0.391 and 0.427, respectively). However, more than half of the patients with MELD scores higher than 11.5 or Child–Pugh classification B died within 5 years after the operation. Furthermore, approximately 90% of patients with MELD scores higher than 11.5 or Child–Pugh classification C died within 10 years after the operation. No similar studies have been reported that allow comparison with our data.

This study has some drawbacks and limitations. First, it is a retrospective study, which leads to an inherent selection bias that cannot be overcome. A randomized investigation is necessary to confirm the conclusions. Second, the cause of death was not determined because only telephone follow-up was performed. Third, we did not consider the effect of antiviral agents in patients with hepatitis B. However, we plan to study this subgroup in the future.

ERCP can be considered an effective and safe therapeutic method in cirrhotic patients with choledocholithiasis regardless of Child–Pugh classification. Sufficient preoperative discussion and preparation for an individual may reduce the risk of complications. MELD scores but not Child–Pugh scores can predict the risk of operative complications.
